# Measuring dlPFC Signals to Predict the Success of Merchandising Elements at the Point-of-Sale – A fNIRS Approach

**DOI:** 10.3389/fnins.2020.575494

**Published:** 2020-11-20

**Authors:** Nadine R. Gier, Enrique Strelow, Caspar Krampe

**Affiliations:** ^1^Faculty of Business Administration and Economics, Chair of Marketing, Heinrich Heine University Düsseldorf, Düsseldorf, Germany; ^2^Faculty of Business Administration and Economics, Chair of Marketing and Sales, Justus Liebig University Gießen, Gießen, Germany; ^3^Shopper Science, Ferrero Deutschland, Frankfurt am Main, Germany; ^4^Consumer Research and Marketing Group, Department of Social Science, Wageningen University & Research, Wageningen, Netherlands

**Keywords:** consumer neuroscience, fNIRS, merchandising elements, point-of-sale, DLPFC, cortical relief effect

## Abstract

The (re-)launch of products is frequently accompanied by point-of-sale (PoS) marketing campaigns in order to foster sales. Predicting the success of these merchandising elements at the PoS on sales is of interest to research and practice, as the misinvestments that are based on the fragmented PoS literature are tremendous. Likewise, the predictive power of neuropsychological methods has been demonstrated in various research work. Nevertheless, the practical application of these neuropsychological methods is still limited. In order to foster the application of neuropsychological methods in research and practice, the current research work aims to explore, whether mobile functional near-infrared spectroscopy (fNIRS) – as a portable neuroimaging method – has the potential to predict the success of PoS merchandising elements by rendering significant neural signatures of brain regions of the dorsolateral prefrontal cortex (dlPFC), highlighting its potential to forecast shoppers’ behaviour aka sales at the PoS. Building on previous research findings, the results of the given research work indicate that the neural signal of brain regions of the dlPFC, measured with mobile fNIRS, is able to predict actual sales associated with PoS merchandising elements, relying on the *cortical relief effect*. More precisely, the research findings support the hypothesis that the reduced neural activity of brain regions associated with the dlPFC can predict sales at the PoS, emphasising another crucial neural signature to predict shoppers’ purchase behaviour, next to the frequently cited *reward association system*. The research findings offer an innovative perspective on how to design and evaluate PoS merchandising elements, indicating fruitful theoretical and practical implications.

## Introduction

The (re-)launch of products is frequently accompanied by point-of-sale (PoS) marketing campaigns, given that effective PoS merchandising elements have been shown to significantly increase sales of advertised products (Sinha and Verma, 2017). Predicting the success of these PoS marketing campaigns in terms of the company’s objectives, for example forecasting the sales before its launch, is of substantial economic importance. An aspect that is reflected in the multibillion-dollar investments companies spend on advertising and merchandising each year (Guttman, 2019). Consequently, a significant amount of research investigated the PoS and its effective design. In this regard, previous PoS research examined in particular the assortment size, the in-store design and the PoS atmosphere. The assortment size and the associated choice overload effects have been investigated most frequently, identifying the circumstances and operating principles in form of an inverted U-shape function between variety and purchase probability (Chernev, 2006; Heitmann et al., 2007; Grant and Schwartz, 2011; Chernev et al., 2012; Beneke et al., 2013). Other research examined PoS in-store demonstrations, product presentations and consumer inspiration, which showed positive effects on attention and evaluation processes of consumers (Nordfält and Lange, 2013; Townsend and Kahn, 2014; Huddleston et al., 2015; Phillips et al., 2015; Bottger et al., 2017). Considering the sensory complexity of the PoS, previous research investigated also the store environments and the PoS atmosphere, exploring how multisensory aspects like music, scent and touch influence shopping behaviour in combined fashion. The results indicate that congruent and matching modalities seem to be most favourable by consumers (Mattila and Wirtz, 2001; Spence and Gallace, 2011; Quartier et al., 2014; Spence et al., 2014; Michel et al., 2017). Although it has been shown that investments in PoS atmospherics and product arrangements can pay off, most merchandising activities are still associated with high costs (Spence et al., 2014). Moreover, many operating stimuli at the PoS that have been shown to greatly influence shoppers are only analysed in isolation without considering the complexity of the entire PoS and its various influencing factors. Consequently, the efficient and effective prediction of the success of PoS marketing campaigns on market level is of great interest for research and practice, given that it might provide a holistic picture of the marketing activities at the PoS that may reduce misinvestments. It is, thus, not surprising that retailers and producers, who launch and promote a myriad of new product variations every year, try to implement marketing campaigns that have been effectively tested before.

The selection of merchandising elements is frequently grounded on insights that are received from exploring the consumers’ perceptions of the – advertised – product or service-associated attributes. In order to measure the consumers’ perceptions of these attributes, self-report measurements are often used, asking consumers directly about their subjective opinions in regard to a product or service. Although self-report measurements have been indicated to be beneficial in some marketing studies, social psychology suggests that self-reports, when used in isolation, are unreliable to accurately predict the consumers’ preferences (Nisbett and Wilson, 1977; De Cremer et al., 2008; Petit and Bon, 2010; Baldo et al., 2015b). This is mostly because the consumers’ expressed intentions do not always translate into actual (purchase) behaviour or even sales (Ajzen, 1991; Padel and Foster, 2005; Frank and Brock, 2018). Against this background, other measurements might be more expedient to solve the indicated matter (Ariely and Berns, 2010; Plassmann et al., 2015; Karmarkar and Yoon, 2016).

The application of neuropsychological methods, using neural brain activity data to forecast products and marketing campaigns success, has been indicated to offer a promising approach to gain further knowledge about the consumers’ perception processes (Ariely and Berns, 2010; Berns and Moore, 2012; Falk et al., 2012, 2015; Plassmann et al., 2015; Venkatraman et al., 2015; Daugherty et al., 2016; Karmarkar and Yoon, 2016; Kühn et al., 2016; Motoki et al., 2020; Tong et al., 2020). Plassmann et al. (2007) explored, for example, how neuropsychological methods could be used to investigate brand equity as a determining factor that influences the perception and, consequently, the behaviour of consumers. Subsequently, multiple studies demonstrated the predictive power of neuropsychological data, displaying the capability of forecasting music and movie success or advertising elasticities of television ads (Baldo et al., 2015a; Boksem and Smidts, 2015; Venkatraman et al., 2015; Cha et al., 2019; Tong et al., 2020). Although the predictive power of neuropsychological methods has been demonstrated to outperform ‘traditional’ marketing methods (Venkatraman et al., 2015), neuropsychological methods and the generated neuropsychological insights are only partially adapted in practice. One reason for this might be that previous research often emphasised *reward associations* in order to predict sales with the utilisation of neuropsychological methods (Ariely and Berns, 2010; Plassmann et al., 2015). Thereby the predictions rely on medially and subcortical located brain regions of the *reward evaluation system*, such as the nucleus accumbens (NAcc), the ventral striatum, the orbitofrontal cortex (OFC) and the ventromedial prefrontal cortex (vmPFC). These brain areas can only be measured with stationary neuroimaging methods, such as functional magnetic resonance imaging (fMRI), whose application is quite costly and time-consuming. However, although just recently a study conducted by Cha et al. (2019) indicated that the application of functional near-infrared spectroscopy (fNIRS) allows to correlate medial prefrontal cortex (mPFC) neural activity to popularity of music on YouTube, another – in previous research often neglected – neural signature might as well be decisive to predicting PoS sales, namely the deactivation of the dorsolateral prefrontal cortex (dlPFC). The dlPFC is known to play a major role in decision-making by integrating cognitive evaluations whilst modulating affective reward responses (Hare et al., 2009). Frequently, increased dlPFC activity is associated with cognitive (self-)control in decision-making and other cognitive processes such as working memory, abstract problem solving and exertion of control in order to favour long-term goals (Miller and Cohen, 2001; Hare et al., 2009; Carlén, 2017). For example, in food-related value-based decision-making increased neural activity in brain areas of the dlPFC have been identified for participants that execute a greater self-control on their food choice (Hare et al., 2009). Simultaneously, a reduced neural activity of the dlPFC has been associated for brand-related decisions that require less strategy-based reasoning (Deppe et al., 2005; Schaefer and Rotte, 2007; Koenigs and Tranel, 2008; Krampe et al., 2018a). First shown in the study by Deppe et al. (2005), decision sets that include the participants favoured brand, emotionalise the choice, which allows a quicker, straightforward and less complex decision-making process in favour of the preferred product, a replicated and robust effect called *cortical relief effect*.

In conclusion, preferred choice options seem to be easier to process, which makes it easier to choose for the favoured product during a decision-making process that seem to be less cognitively controlled and assumed to elicit a reduced activity in brain regions of the dlPFC (Deppe et al., 2005; Schaefer and Rotte, 2007; Koenigs and Tranel, 2008; Krampe et al., 2018a). Less self-controlled decisions might, therefore, result in more impulsive decision-making, choosing the option that is preferentially presented in a choice situation (Boettiger et al., 2007; Kable and Glimcher, 2007; Hare et al., 2009). Consequently, merchandising elements that are about to expose a reduced neural activity in brain regions ascribed to the dlPFC might be less cognitively engaging, resulting in more impulsive decisions, which might rescale in increased sales at the PoS. Hence, while earlier neuropsychological studies that aimed to predict consumer behaviour on population level with neuropsychological methods focussed mainly on medial and subcortical located brain regions of the *reward evaluation system*; only a few studies considered the dlPFC in their prediction models. Consequently, this research work is one of the first to evaluate whether the reduced neural dlPFC activity, as a neural signature, can predict PoS sales, building on insights of the *cortical relief effect*.

Having this in mind, the current research work aims to explore the predictive power of the cortical brain regions of the dlPFC to forecast the success of PoS merchandising elements. By doing so, the given research work overcomes the limitations of stationary neuroimaging methods by utilising mobile fNIRS as a portable applicable neuropsychological method for the research field of shopper neuroscience, demonstrating its potential application in ecological valid setting, such as the PoS (Kopton and Kenning, 2014; Çakir et al., 2018; Krampe et al., 2018b). Against this background, the given research work aims to explore whether mobile fNIRS – as a mobile applicable neuroimaging method – has the potential to predict the success of PoS merchandising elements by rendering significant neural *cortical relief* signatures of the dlPFC.

## Predicting Success of PoS Merchandising Elements – the ‘Duplo’ Case

**FIGURE 1:**
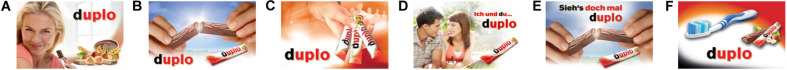
Merchandising elements of the product ‘duplo’. The six merchandising elements were used in prior studies (Kühn et al., 2016; Strelow and Scheier, 2018) and the current study, including: **(A)** a *woman* eating a ‘duplo’ bar, used at the PoS from 1995 to 2015; **(B)** *hands* holding a ‘duplo’ bar, representing a TV campaign that had been on air for 6 months from 2011 to 2012; **(C)** a *group* of people and three ‘duplo’ bars, which represented a TV campaign that had been on air for nearly 20 years between 1991 and 2010; **(D)** a *couple* with a ‘duplo’ bar and **(E)** *hands* holding a ‘duplo’ bar *with text*, which were not used in advertising previously, as well as **(F)** a *toothbrush* with a ‘duplo’ bar used as control merchandising element. Figure adapted from Kühn et al. (2016). Permission to reuse has been obtained.

A special case in the analyses of PoS merchandising elements is the product ‘duplo’ by Ferrero (Ferrero Deutschland GmbH, n.d.). ‘Duplo’ constitutes a special case for research, since its effects on shoppers’ processing and behaviour were not only explored in prior studies with neuropsychological and traditional marketing methods (Kühn et al., 2016; Strelow and Scheier, 2018; Strelow et al., 2020), allowing comparisons between different data types, but also provide unique, real-market stimuli materials for research, that are, in contrast to research stimuli specifically designed for a study, highly ecologically valid. The product ‘duplo’ was introduced to the German market in 1964 and is currently the market leader of chocolate bars in Germany, with a turnover of 200 million Euro (VuMA, 2019b). There, more than 50% of the turnover is achieved by secondary (out of shelf) displays, which are displayed with PoS merchandising elements (Briesemeister and Selmer, 2020). Over the past 40 years, many PoS merchandising elements have been used to promote the chocolate bar. Six merchandising elements were explored by prior research, representing a typical choice set for marketing campaigns, including past and recent PoS and TV campaigns as well as similar but unknown merchandising elements (Figure 1).

**FIGURE 2:**
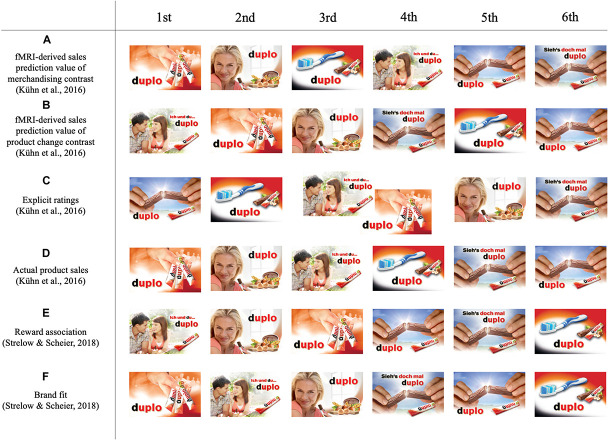
Ranking of the six merchandising elements based on prior research. Ranking order of the merchandising elements derived from: **(A)** fMRI-derived sales prediction value of merchandising elements from Kühn et al. (2016); **(B)** fMRI-derived sales prediction value of product contrasts from Kühn et al. (2016); **(C)** the explicit rating of participants of the study by Kühn et al. (2016); **(D)** actual product sales of the field study of Kühn et al. (2016); **(E)** mean average reward association strength by Strelow and Scheier (2018); **(F)** brand-fit score of reward association by Strelow and Scheier (2018). Figure adapted from Kühn et al. (2016) and Strelow and Scheier (2018). Permission to reuse has been obtained.

An fMRI study conducted by Kühn et al. (2016) investigated the different PoS ‘duplo’ merchandising elements on neural level. In particular, two fMRI-derived sales prediction values were extracted based on the neural BOLD signals measured (1) during the perception of the merchandising elements contrasted to the implicit baseline and (2) for the signal change from the baseline contrast of (the advertised) package ‘duplo’ product seen before and after the merchandising element. The fMRI-derived sales prediction values summarised the signal of multiple neural regions, whereby the prediction was mainly driven by the neural activity of the *reward system* (NAcc and medial OFC) and the *deactivation of the dlPFC* (Brodmann area 9 and 46). Furthermore, explicit subjective ratings of the ‘duplo’ merchandising elements were evaluated. In order to measure the actual sales – defined as the revenue generated by the different merchandising elements – the merchandising elements were tested at the PoS in a field experiment in parallel to the fMRI study (for detailed information, please see Kühn et al., 2016) (Figure 2D). Results demonstrated that the fMRI-derived sales prediction value based on the merchandising element presentation was the best predictor for the sales numbers (Figure 2A). While the first two and last two ranking positions were equivalent between fMRI-derived sales prediction value of merchandising elements and actual sales, only one match at the third position was found for the subjective rankings (Figure 2C) and no match for the fMRI-derived sales prediction value of the product contrast (Figure 2B). Inspecting the integrated neural brain areas *ad hoc* in detail, Kühn et al. (2016) identified the medial OFC as most predictive for actual market sales.

In order to explore the shoppers’ associations with the different PoS merchandising elements and to understand the shopper response to the merchandising elements, following the fMRI study, the merchandising elements were examined in a second study conducted by Strelow and Scheier (2018), utilising an implicit reward association test (IAT). During the IAT, each PoS merchandising element as well as the brand itself were assessed on different reward values that were spontaneously associated with the brand and the merchandising element. From the results of the IAT for the merchandising elements, Strelow and Scheier were able to discriminate the lower three merchandising from the top three merchandising elements, although the ranking order was not congruent with the actual sales numbers identified by Kühn et al. (2016) (Figure 2E). Subsequently, the fit between the merchandising elements and the brand’s reward associations was analysed, indicating that the first and last two ranks of the actual PoS sale performance can be determined by the data (Figure 2F). The fit of the brand associations with the merchandising element associations can be interpreted either as an *enhancement* or at least as a confirmation of the brand reward associations representing the degree of congruence between the expected associations elicited by the brand and the associations evoked by the brands merchandising elements.

In conclusion, data from both (neuro)psychological methods, the fMRI data and the IAT data, seem to outperform self-report shoppers’ ratings of the merchandising elements. A high brand-fit score as indicated by Strelow and Scheier (2018) between the merchandising element and the brand seems to be predictive for the success of a merchandising element, since the shoppers’ expected and experienced brand associations are congruent with the merchandising element, potentially resulting in a *cortical relief effect*, reducing the experienced cognitive dissonance. In the study conducted by Kühn et al. (2016) the fMRI-derived sales prediction value based on the merchandising element presentation were most predictive for actual sales data. Although, the brain regions of *reward evaluation system*, especially medial OFC, were again highlighted as the driving force for the prediction, a decreased neural activity in the dlPFC was integrated in the formula to predict sales, an aspect that represents reduced cognitive effort and greater *cortical relief* (MacPherson et al., 2002; Carter and van Veen, 2007; Cho et al., 2010; Izuma et al., 2010; Bartra et al., 2013). Building on previous research, which demonstrated that mobile fNIRS is particularly capable of measuring neural cortical activity, especially lateral areas of the prefrontal cortex (Krampe et al., 2018a; Liu et al., 2018), the investigation of the neural signatures of the dlPFC’s deactivation might be a fruitful avenue to predict the success of merchandising elements. While doing so, this research work opens up the potential application of mobile fNIRS in a realistic shopping environment, namely the PoS, to predict success on market level. Hence, the given research work aims to explore, whether the dlPFC can act as a predictive neural signature for actual market sales by utilising and validating mobile fNIRS as a mobile neuropsychological method for the research field of shopper neuroscience, leading to the following hypothesis:

The neural signatures of the dlPFC during the perception of merchandising elements measured with mobile fNIRS are able to predict the sales associated with the PoS merchandising elements.

## Materials and Methods

### Participants

In line with previous research (Rampl et al., 2012; Kühn et al., 2016; Krampe et al., 2018a,b; Strelow and Scheier, 2018) only healthy, female participants (*N* = 45), who indicated that they were mainly responsible for the grocery shopping in their household, were recruited to participate in this study. Female participants were recruited because women are more frequently responsible for the household’s grocery shopping (VuMA, 2019a,c,d,e; BVE, 2020). Due to bad signal quality, 12 participants had to be excluded from the data analysis, resulting in a final sample size of *n* = 34 (*M*_*age*_ = 41.06, *SD*_*age*_ = 8.41; Age_*min*_ = 23, Age_*max*_ = 54). All participants were right-handed and had no history of major psychological or neurological disorders.

### Experimental Task Procedure

After participants were welcomed, they were informed verbally and in written form about the aim of the study, the task and the utilised mobile fNIRS device. Once participants fully understood the task, a written informed consent was signed in accordance with the Declaration of Helsinki. Thereafter, participants were seated in front of a computer screen and the mobile fNIRS headband was attached on the participants forehead. In order to increase consistency between the participants measured brain regions, the mobile fNIRS headband was locally standardised on the vertical axis using the craniometric point of the nasion as an orientation point and the middle of the two preauricular points for positioning on the horizontal axis, covering the prefrontal cortex. Before starting the experimental task, data quality was checked and, if necessary, signal quality was improved by shifting the hair away from the detectors, making direct skin contact. In addition, the fNIRS headband was covered with an light-protecting cap to control for external light sources. Once the preparation was finished, participants were instructed to look at the computer screen while the task was performed.

**FIGURE 3:**
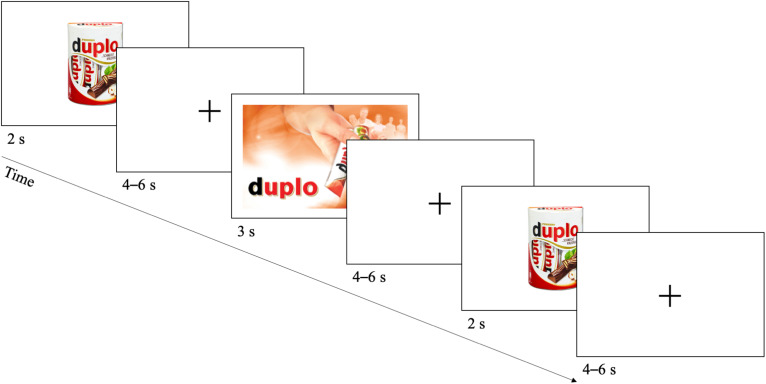
Schematic representation of a trial in the experimental task. The task design is adapted from Kühn et al. (2016). During each trial, one merchandising element was displayed randomly for 3 s. Before and after the merchandising element, the advertised product was shown for 2 s. All stimuli were separated by a randomized jitter of 4–6 s. Figure adapted from Kühn et al. (2016). Permission to reuse had been obtained.

The task was designed analogous to the paradigm developed by Kühn et al. (2016) (Figure 3), applying an event-related experimental design. During the task, a merchandising element was displayed for 3 s, followed by a randomised jitter of 4–6 s. Before and after the merchandising element, the advertised product was shown for 2 s, again followed by a randomised jitter of 4–6 s. In total, every merchandising element was shown six times, whereby the order of the merchandising elements was totally randomised. The task was performed twice, resulting in a total number of 72 trials, with 12 trials for every of the six merchandising elements. After completing the task, the mobile fNIRS device was removed and participants were asked to complete a final questionnaire, assessing demographics as well as their explicit subjective ranking of the merchandising elements. At the end of the study and a verbal disclosure, participants received a monetary incentive for their participation and were free to leave.

### fNIRS Data Collection

**FIGURE 4:**
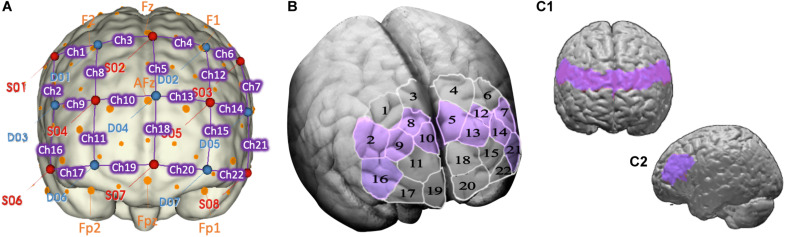
fNIRS optode montage setup (topolayout) with marked regions representing Brodmann area 9 and 46. **(A)** fNIRS optode montage setup of the sources (S; red) and detectors (D; blue) with the associated fNIRS channels (Ch; purple) and the coordinates of the EEG 10-20 system (orange dots) (modified graphic from Nissen et al., 2019), **(B)** fNIRS channel areas plotted on a standardised brain with channels constituting Brodmann area 9 and 46 marked in purple (modified graphic from Krampe et al., 2018b), **(C1)** Brodmann area 9 and **(C2)** Brodmann area 46 marked in purple.

The continuous-wave fNIRSport-System (NIRx Medical Technologies, Berlin, Germany) was used for data collection (Boas et al., 2014; Scholkmann et al., 2014). In general, fNIRS measures cerebral haemodynamic responses through near-infrared light sources (Ferrari and Quaresima, 2012). The mobile fNIRS system recorded optical signals on two-wavelengths (760 and 850 nm) at a sampling rate of 7.81 Hz. As imaging depth increases with emitter-detector distance, but signal quality is suggested to be best at a separation of 3 cm, the optodes and diodes are set to the distance of 3 cm (McCormick et al., 1992; Gratton et al., 2006; Ferrari and Quaresima, 2012; Gagnon et al., 2012; Naseer and Hong, 2015). The system consists of 22 channels, comprising eight light sources and seven detectors (Figure 4). In order to identify the equivalent brain areas of Brodmann area 9 (Figure 4C1) and 46 (Figure 4C2), the dlPFC definition had to be transferred to the mobile fNIRS optode montage setup (Figure 4A). Channels classified as relevant to cover Brodmann area 9 are Ch2, Ch5, Ch7, Ch8, Ch9, Ch10, Ch12, Ch13, and Ch14, and for Brodmann area 46 are Ch16 and Ch21 (Figure 4B). The NIRS-Star software package (version 14.2) was used for checking signal quality and data collection.

The valid application of mobile fNIRS in the field of consumer and shopper neuroscience has been demonstrated in several studies (Kopton and Kenning, 2014; Çakir et al., 2018; Krampe et al., 2018a,b). Most of the consumer neuroscience research using fNIRS focussed on the identification of neural correlates associated with merchandising in virtual in-store settings (Krampe et al., 2018b; Liu et al., 2018) or used fNIRS measurements to predict individual food-choice behaviour (Çakir et al., 2018). A recent fNIRS study conducted by Cha et al. (2019) correlated neural activation patterns of the mPFC to online popularity of pop music on YouTube, presenting an extension of earlier studies that predicted music popularity in the field of consumer neuroscience applying fMRI (Berns and Moore, 2012). Overall, prior fNIRS research suggested that especially cortical regions are measurable, whilst brain regions located medially within in the brain or subcortically are not assessable with mobile fNIRS (Krampe et al., 2018a). Furthermore, most of previous fNIRS studies focussed on the medial brain regions, with only one study correlating neural activity pattern to behaviour on population level. As a result, the predictive value of lateral brain areas has not yet been addressed and mobile fNIRS as an innovative neuropsychological method in the field of consumer and shopper neuroscience, requiring further profound and robust validation.

### fNIRS Data Analysis

In order to analyse the collected data, data was pre-processed using the NIRx Software Package (NIRx Medical Technologies, Berlin, Germany). In order to increase signal quality, channels exhibiting discontinuous shifts during the measurement were removed. Furthermore, fNIRS data time series were smoothed, applying a band-pass filter (high and low frequency filter) (Naseer and Hong, 2015; Pinti et al., 2019) with the frequently applied low cut-off frequency of 0.01 Hz and high cut-off frequency of 0.2 Hz (Franceschini et al., 2003; Hu et al., 2012; Spichtig et al., 2012; Krampe et al., 2018a; Nissen et al., 2019) in order to control for physiological noises and artefacts such as heartbeat and Mayer waves (Scholkmann et al., 2014; Naseer and Hong, 2015; Pinti et al., 2019). The modified Beer-Lambert law was used to convert raw light absorption rates into haemoglobin concentrations (Kocsis et al., 2006; Kopton and Kenning, 2014; Scholkmann et al., 2014). Haemodynamic states were computed in accordance with commonly used pathlength factors (for 750 nm set to 7.25 and for 850 set to 6.38) (Essenpreis et al., 1993; Kohl et al., 1998; Zhao et al., 2002). For the further analysis only oxygenated haemoglobin signals were interpreted, as they seem to better correlate with cerebral blood flow (Hoshi et al., 2001). Information on the oxygenated haemoglobin concentrations are available in the Supplementary Material.

A general linear model (GLM) was set up for every participant and convolved with the haemodynamic response function, including six regressors with one for each merchandising element and an additional 12 regressors for the product stimuli (six before and six after each merchandising element). The GLM was first calculated on a single subject individual level (within-subjects level), and subsequently, a second-level group contrasts analysis was carried out to calculate neural activations across subjects (between-subjects level). In order to extract standardised activation values, a *t*-contrast was executed for each merchandising element against the implicit baseline, using the *t*-values in the further analysis. Given that significant activation differences are not of interest, the contrast analysis was used as a procedure to standardise the neural activations, which made a multiple comparison correction redundant. To test the hypothesis, fNIRS-derived sales prediction values were calculated from the standardised activation values of the *t*-contrasts for every merchandising element, respectively (Equation 1). The resulting fNIRS-derived sales prediction values can be interpreted according to their degree of reduced dlPFC neural activity. Hence, the fNIRS-derived sales prediction values for Brodmann area 9 and 46 were used to rank the order of the merchandising elements from lowest to greatest values, whereby a greater neural deactivation (more negative value) corresponds to a higher rank. Thus, the ranking is a result of the least neural activity, displaying less cognitive interfered processing (*cortical relief effect*) that is hypothesised to translate to sales at the PoS. Consequently, the resulting rank order based on the reduced dlPFC signal values should coincides with the rank order of the actual sales data. In order to evaluate the predictive success of the fNIRS-derived sales prediction values rankings with the original sales data, the results were compared qualitatively and based on Spearman rho correlation coefficients for the ordinal rank orders as well as on Pearson correlation for the quantifiable sales prediction values and actual sales data at a significance threshold of *p* < 0.05.

**EQUATION 1:**
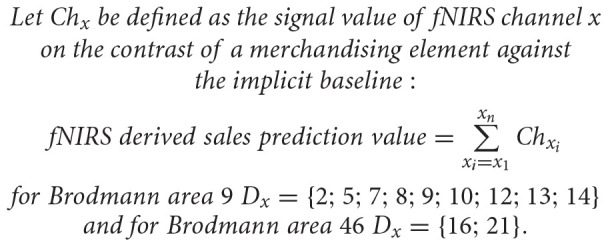
Formula for fNIRS-derived sales prediction value. The *t*-values of channel Ch2, Ch5, Ch7, Ch8, Ch9, Ch10, Ch12, Ch13, and Ch14 were allocated to represent Brodmann area 9, while for Brodmann area 46 the channel Ch16 and Ch21 were defined. This calculation was performed for each merchandising element, resulting in six fNIRS-derived sales prediction values per Brodmann area (9 and 49).

Conclusively, based on the neural data analysis two different types of dlPFC fNIRS-derived sales prediction values were extracted and rank ordered, according to their degree of the reduced dlPFC activity. First, the fNIRS-derived sales prediction values of Brodmann area 9; and second of Brodmann area 46, calculated from the contrasts of each merchandising element against the implicit baseline, have been evaluated. The participants’ explicit subjective rating of the merchandising elements was also evaluated, whereby the total number of 1st rank positions for each merchandising element was taken as an indicator. Finally, and in order to estimate the predictive power of the different data types, the actual sales associated with the merchandising elements – defined as the revenue generated by the different merchandising elements – were adopted from Kühn et al. (2016), who explored the revenues generated by the merchandising elements on a quarter display at the PoS in a supermarket (for detailed information on data, data collection and analysis, please see Kühn et al., 2016).

## Results

**FIGURE 5:**
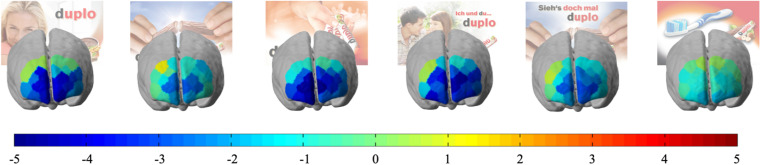
*T*-value coloured activation maps for the contrast of merchandising element against the implicit baseline. The associated merchandising element is displayed behind the brain map. Channel allocation can be found in Figure 4B. Colour bar indicates the *t*-values of the contrasts.

**FIGURE 6:**
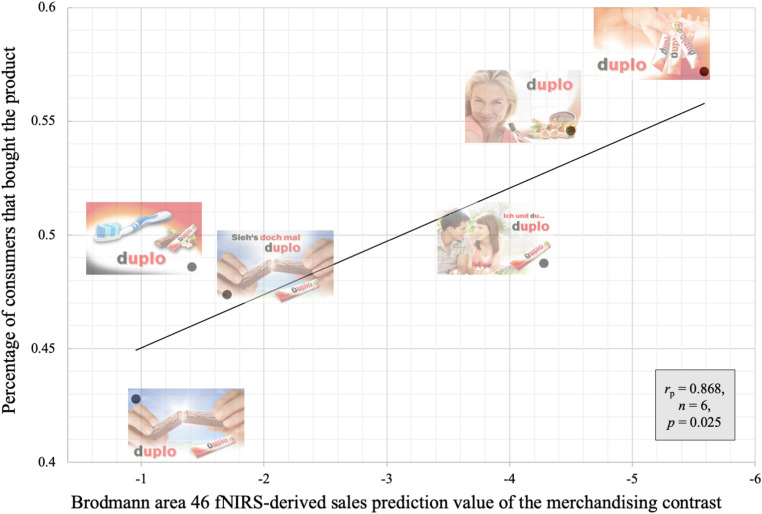
Scatterplot depicting the association between the Brodmann area 46 fNIRS-derived sales prediction value of the merchandising contrast, and actual product sales (Kühn et al., 2016) expressed in percentage of the customers that bought the product on the display with the merchandising element. Pearson correlation presented in the grey box.

**FIGURE 7:**
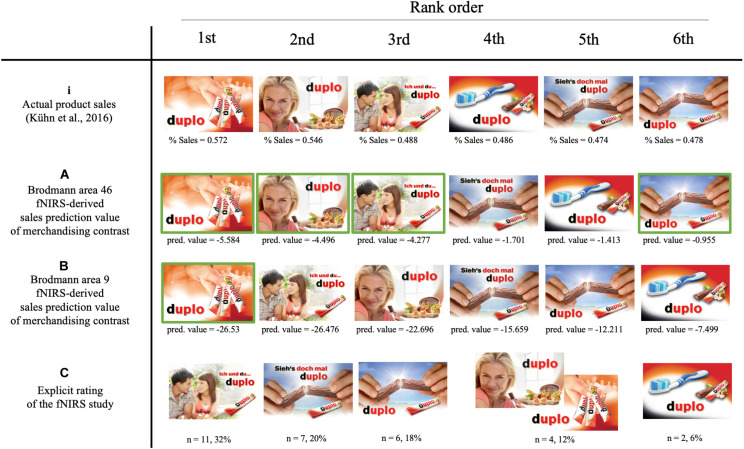
Ranking of the six merchandising elements. **(i)** The rank order based on actual sales data from Kühn et al. (2016). Rank order of the merchandising elements derived from fNIRS-derived sales prediction value of **(A)** Brodmann area 46 and **(B)** Brodmann area 9 as well as the **(C)** explicit subjective rating of the participants in the fNIRS study. The fNIRS-derived sales prediction values and percentages are displayed underneath the merchandising element. Matched rank order positions are marked in red. Figure partly adapted from Kühn et al. (2016). Permission to reuse has been obtained.

Supporting the hypothesis, the results suggest that the neural sales prediction values of brain regions of the dlPFC calculated from the merchandising contrasts (Figure 5) are able to predict the actual sales associated with PoS merchandising elements. The best predictor is the fNIRS-derived sales prediction values of Brodmann area 46. This finding was confirmed by the correlation analyses that revealed a positive significant Spearman rho correlation on the rank order data (*r*_*s*_ = 0.943, *n = 6*, *p* = 0.005) and a positive significant Pearson correlation on the sales prediction values and actual sales (*r*_*p*_ = 0.868, *n = 6*, *p* = 0.025) (Figure 6). For the qualitatively comparisons with the actual sales data ranking (Figure 7i), this rank order has all rank positions matched with the exception of the last 4th and 5th positions, which are reversed (Figure 7A).

Similarly, the neural results reveal that the first rank position based on the calculated Brodmann area 9 fNIRS-derived sales prediction value of the merchandising contrast (Figure 7B) corresponds to the rank positions of the actual sales data. However, the associated correlations on rank order and sales prediction value with the actual sales data failed to reach significance threshold of *p* < 0.05 (*r*_*s*_ = 0.771, *n* = 6, *p* = 0.072; *r*_*p*_ = 0.648, *n* = 6, *p* = 0.164). For the explicit subjective ranking no matched rank positions could be identified qualitatively (Figure 7C), confirmed by small, non-significant correlations with the actual sales data (*r*_*s*_ = −0.29, *n* = 6, *p* = 0.577; *r*_*p*_ = 0.309, *n* = 6, *p* = 0.551). The *t*-values on each channel and scatterplots on the non-significant predictors are available in the Supplementary Material. Thus, fNIRS-derived sales prediction values aggregating the channels constituting Brodmann area 46 could resample the actual sales data best.

## Discussion

The current research work aims to explore the predictive power of brain regions ascribed to the dlPFC to forecast the success of PoS merchandising elements, thereby validating mobile fNIRS – as a portable applicable neuropsychological method – and opening up its potential application in realistic shopping environments, such as at the PoS. As one of the first studies, this research work evaluates the neural signatures of the dlPFC deactivation in isolation to predict market sales success with mobile fNIRS, building on the *cortical relief effect*. More precisely, the integration of mobile fNIRS in the field of shopper neuroscience has been used to investigate six PoS merchandising elements, which have been examined with marketing methods in earlier studies, while overcoming the limitations associated with stationary neuroimaging methods (Kühn et al., 2016; Strelow and Scheier, 2018). The research findings support the hypothesis that the deactivation of the dlPFC is predictive for the shopper behaviour aka sales at the PoS, highlighting an additional crucial neural signature measurable with mobile fNIRS. The results show that fNIRS-derived sales prediction values of Brodmann area 9 and 46 are capable of predicting the actual sales of PoS merchandising elements, whereby Brodmann area 46 (consisting of channels 16 and 21) seem to be the most predictive brain area of the dlPFC.

In the context of prior studies on the ‘duplo’ case, the current research findings suggest that merchandising elements promoting a brand are processed in two neural signatures of the (prefrontal) cortex, leading to different cognitive processes. Whereas in the past the neural activity of the *reward evaluation system* has been used to predict marketing, advertising and sales effects at the PoS, the role of *cortical relief effects* and reduced cognitive controlled processes have been neglected. Although occasionally studies integrated the dlPFC besides other brain regions in their prediction models, cortical relief processes have – to the best of the authors’ knowledge – not yet been used to predict and explain purchase behaviour at the PoS.

Supposing that 70% of the purchases at the PoS are spontaneous and given that an act of purchase takes approximately about 60 s (Hertle and Graf, 2009; Valizade-Funder and Heil, 2010), it is suggested that an habituative, less self-controlled process takes place in most of the purchases (Rook and Fisher, 1995). Consequently, any kind of irritation that disrupts the state of cortical relief by incongruency or aspects that require more cognitive effort could potentially interrupt the act of impulsive purchase, resulting in a termination or, at least, a delay in the cognitive or affective purchase process of shoppers. This effect seems to be particularly relevant when shoppers experienced a conflict between their perceived brand image and the triggered reward associations elicited by the PoS merchandising element – a neuropsychological process, which seem to result in an increased neural cortical dlPFC activity (Deppe et al., 2005; Plassmann et al., 2007; Koenigs and Tranel, 2008; Kato et al., 2009; Krampe et al., 2018b) and which could be measured with mobile fNIRS. Likewise, the congruency of the brand image and the associated PoS merchandising element might result in a neuropsychological (*cortical) relief effect* for congruent brand-merchandising PoS elements or vice versa result in an increased neural activity effect in the dlPFC, when the product and merchandising element are perceived as incongruent. Both effects can, consequently, be measured in brain regions of the dlPFC, indicating its specificity to predict sales at the PoS. Consequently, next to the *reward association system*, brain regions of the dlPFC might also function as a process variable to predict sales in a PoS setting. The utilisation of mobile fNIRS with its technical capabilities to measure cortical brain regions might, therefore, provide an innovative and fruitful method for future research.

### Implications

The research findings provide several implications for marketing theory and practice. First, from a theoretical perspective, the research findings suggest that the shopper behaviour at the PoS is not only driven by reward associations offered by brands, but is also influenced by the perceived (in-)congruency and the level of conflicts or cortical relief experienced between the shoppers’ brand image and the experienced PoS merchandising element. While earlier neuropsychological studies investigated mainly medial and subcortical located brain regions of the reward evaluation system to forecast population success; only a few studies considered the dlPFC to predict shoppers’ behaviour. Consequently, this research work is one of the first that evaluates the predictive power of brain regions ascribed to the dlPFC neural deactivation, providing an innovative approach to interpret consumer responses to merchandising elements at the PoS.

Second, as a methodological contribution, the validation of a mobile and in its application fast-growing methodology of mobile fNIRS demonstrates its potential to predict success in real-world settings such as the PoS. Due to its mobile application it provides a great variety of application options for research and practice to measure shoppers’ neural responses directly in complex settings such as the PoS, increasing the ecological validity of research results.

From a practical point of view, the research results offer an innovative perspective on how to design, evaluate or forecast the success of PoS merchandising elements in combination with the to-be-advertised products – including all kind of merchandising elements such as lighting, furnishing, display screens, price tags and information displays. Cortical relief disrupting conflicts can arise on all levels of the customer journey, beginning with the perception of a stimulus and ending in cognitive overload effects elicited by, for example, the overwhelming assortment in the shelves. To carefully match the shoppers’ brand image with PoS merchandising elements in order to reduce conflicts and cognitive dissonance might, consequently, be of high value for producers and retailers. The integration of the idea to investigate the (in-)congruency and potential conflicts as well as its repercussions enables the analysis of the shoppers’ PoS journey by evaluating different merchandising elements, with its aim to reduce or at best avoid conflicts in the perception of the product specific attributes (e.g., the brand image) and the PoS merchandising elements to be used. A comprehensive investigation of all cues that appear at the PoS during a customer journey, to explore all potential reactions of the shoppers’ brain during a shopping trip, to identify cues that potentially reduce the overall net-incongruence at the PoS, might be beneficial. The neuropsychological neuroimaging method of fNIRS may, therefore, be of particular interest as it enables the investigation of the hypothesised effect directly at the PoS because of its mobile, ecological valid usability. Following from this, the research results might be used to explore different PoS merchandising elements to quantify the cognitive engagement represented by the neural activity of the dlPFC evoked by a shopping trip, measured with the use of mobile fNIRS. The ultimate goal would be a measurement of all rewarding and conflicting cues during an average shopping trip, possibly enhanced by the identification of additional motivating cues, to generate a deeper understanding of the shoppers’ behaviour at the PoS.

### Limitation and Future Research Suggestions

One aim of the research work is to indicate the usefulness of mobile fNIRS to predict shopper behaviour at the PoS. The current study provides a first step to actually measure shoppers’ neural activity, when confronted with PoS merchandising elements and products at the PoS, using mobile fNIRS. Nevertheless, this research work investigates the neural signatures on basis of a laboratory setting with an experimental paradigm performed in front of a computer screen. The next logical step for future studies should be to explore whether the research findings received under laboratory settings remain also valid in a naturalistic environment measurement at the PoS, utilising mobile fNIRS in realistic PoS settings. Furthermore, mobile fNIRS is a relative innovative neuroimaging method, at least for the research field of shopper neuroscience, indicating the need to consider the continuous development of its technical capabilities. Future research might, thus, use other more advanced mobile fNIRS devices to improve data quality and reduce the application costs. Finally, whilst interpreting the neural activity and the neural reactions associated with PoS merchandising elements, it is implicitly assumed that the *cortical relief effect* is measured. However, it might be that the merchandising elements have been seen in a TV or PoS campaign before, leading to the measurement of a familiarity effect. This effect might be evoked because the familiar merchandising element might require less cognitive effort to be processed, resulting in a reduced neural activity of the dlPFC. In order to cope with this potential limitation, future studies might replicate the given study with only novel PoS merchandising elements that vary in the degree of their brand fit.

## Conclusion

Whereas previous research work mainly focused on the *reward association system* and its associated subcortical brain regions to predict sales, utilising stationary neuroscientific methods (e.g., Berns and Moore, 2012; Venkatraman et al., 2015; Tong et al., 2020), the research findings of the current study not only suggest that the shoppers’ reward associations seem to be predictive for sales at the PoS, but indicate the importance of the conflicts perceived by the shopper and the congruency between the perceived brand image and the displayed PoS merchandising elements. In other words, the research results signify that the brand ‘duplo’ activates expectation of rewards, which either fits with the associations triggered by the merchandising PoS element or do not fit with the brand’s image perceived by shoppers, leading to either conflicting or supporting, cortical relief effects, displayed by an increase neural activity or a decreased neural activity of the dlPFC, respectively. These neuropsychological processes can, therefore, be quantified with the measurement of the neural activity of the dlPFC, using mobile fNIRS. Consequently, the quantified neural activity of the dlPFC, indicating the congruence between the brand’s image and the triggered reward associations of the PoS merchandising element, might, next to the reward association system, be decisive for the prediction of sales at the PoS, acting as an additional process variable, measurable with mobile fNIRS.

## Data Availability Statement

The datasets presented in this article are not readily available because it was ensured to the participants that their data is not available for third parties and it was guaranteed that participants can request the complete deletion of their datasets at any time. Requests to access the datasets should be directed to nadine.gier@hhu.de.

## Ethics Statement

Ethical review and approval was not required for the study on human participants in accordance with the local legislation and institutional requirements. The patients/participants provided their written informed consent to participate in this study.

## Author Contributions

NG conducted the study, performed the data analysis and wrote the manuscript. NG and CK contributed to the conception and design of the study. NG, ES, and CK were involved in the data collection. ES and CK substantiated sections of the manuscript. All the authors contributed to manuscript revision, read, and approved the submitted version.

## Conflict of Interest

The authors declare that this study received funding from Ferrero Deutschland GmbH. The funder was not involved in the study design, data collection and analysis, interpretation of data, the writing of this article or the decision to submit it for publication. This research work was conducted in cooperation with Ferrero Deutschland GmbH to which ES is directly associated. The authors ensure that the cooperation has in no possible way influenced the research results nor the development of the manuscript.
