# Supplementary figures and images for: Measuring dlPFC Signals to Predict the Success of Merchandising Elements at the Point-of-Sale – A fNIRS Approach
Source: Front Neurosci. 2020 Nov 20;14:575494. doi: 10.3389/fnins.2020.575494 (PMC7714758; doi:10.3389/fnins.2020.575494)

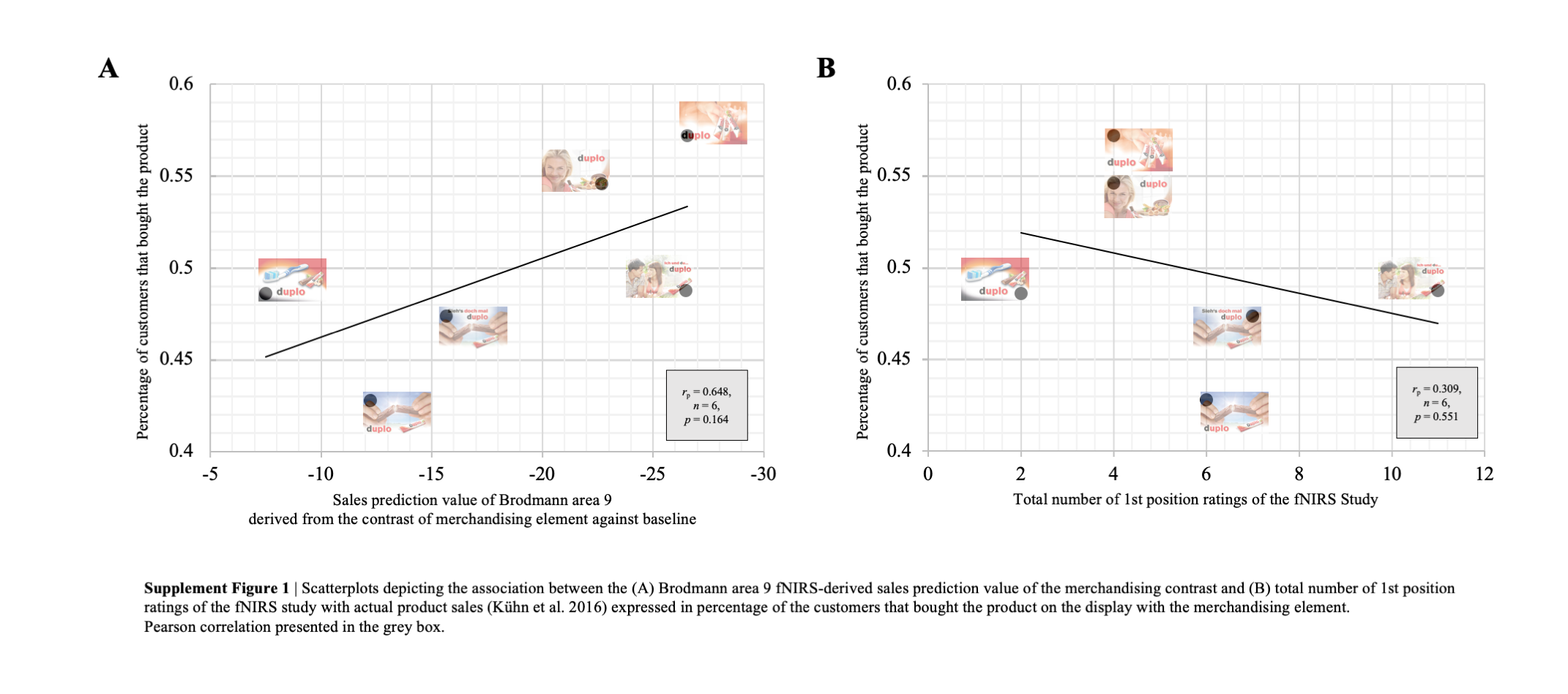

Supplement: Supplementary file 1 [file Image_1.tiff]

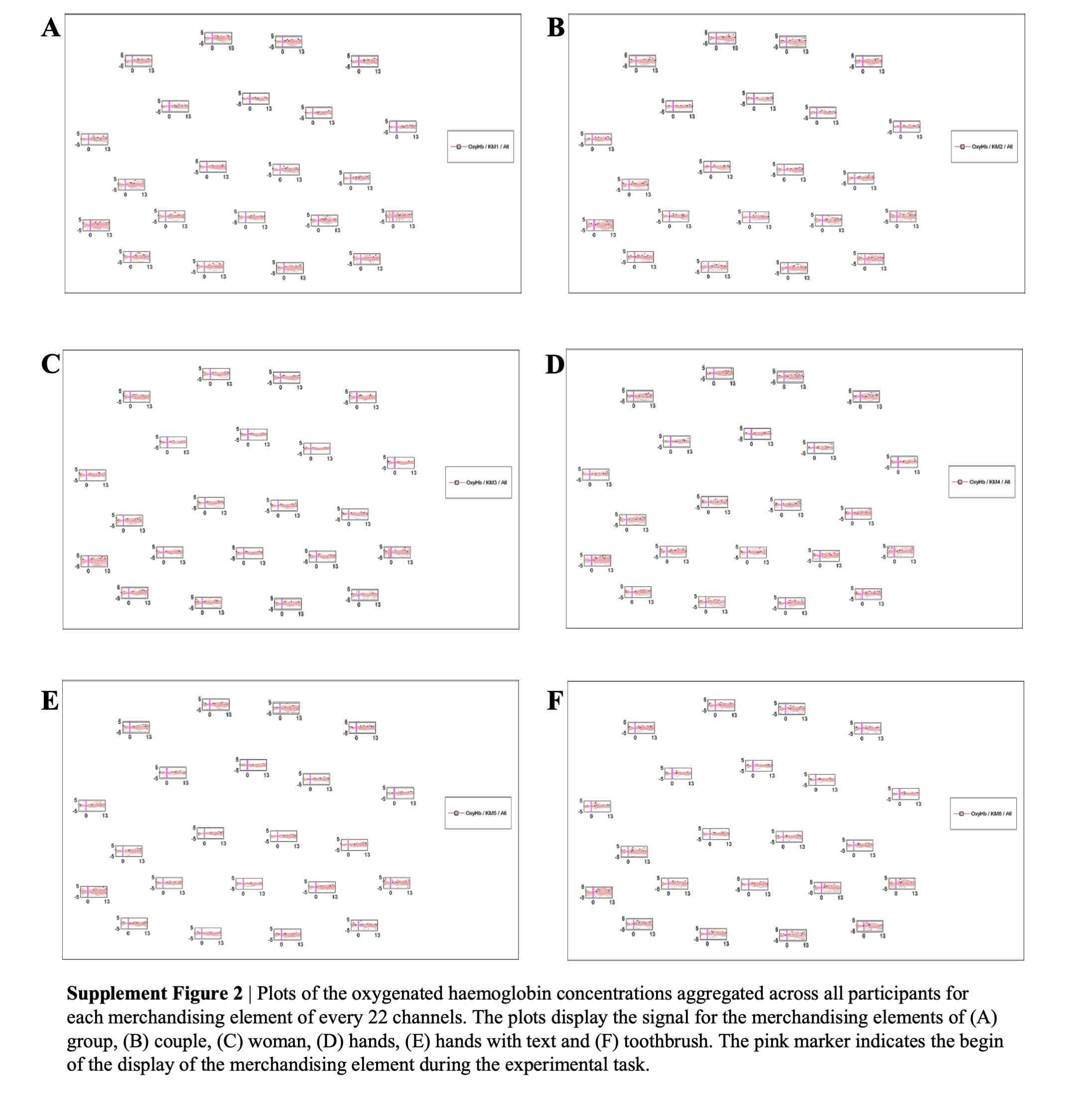

Supplement: Supplementary file 2 [file Image_2.jpg]

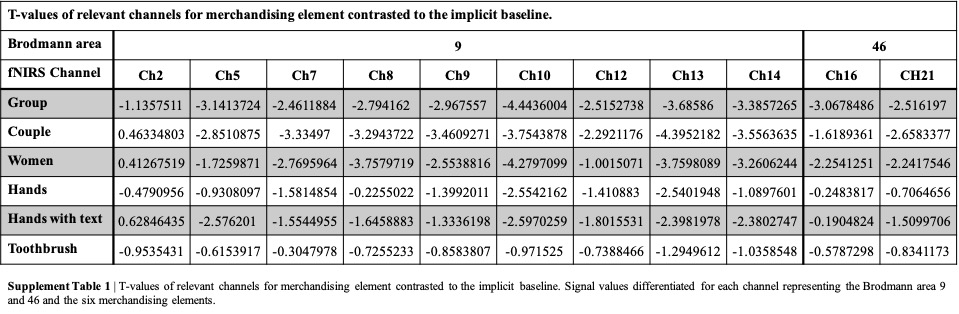

Supplement: Supplementary file 3 [file Image_3.JPEG]
